# Comparative efficacy and safety of the fixed versus unfixed combination of latanoprost and timolol in Chinese patients with open-angle glaucoma or ocular hypertension

**DOI:** 10.1186/1471-2415-11-23

**Published:** 2011-08-19

**Authors:** Jia-Liang Zhao, Jian Ge, Xiao-Xin Li, Yu-Min Li, Yao-Hua Sheng, Nai-Xue Sun, Xing-Huai Sun, Ke Yao, Zheng Zhong

**Affiliations:** 1 Chinese Academy of Medical Sciences, Peking Union Medical College Hospital, Beijing, China; 2 Zhong Shan Ophthalmic Center, Sun Yat-Sen University, Guangzhou, China; 3 Beijing University People's Hospital, Beijing, China; 4 Zhe Jiang University, 1st Hospital, Hangzhou, China; 5 Shanghai Jiaotong University, Xin Hua Hospital of Medical School, Shanghai, China; 6 Xi'an Jiao Tong University, 2nd Hospital, Xi'an, China; 7 Eye & ENT Hospital of Fudan University, Shanghai, China; 8 Zhe Jiang University, 2nd Hospital, Hangzhou, China; 9 Medical Affairs, Pfizer Investment Co. Ltd., China

## Abstract

**Background:**

A noninferiority trial was conducted to evaluate the efficacy of a single evening dose of fixed-combination latanoprost 50 μg/mL and timolol 0.5 mg/mL (Xalacom^®^; LTFC), in Chinese patients with primary open-angle glaucoma (POAG) or ocular hypertension (OH) who were insufficiently controlled on β-blocker monotherapy or β-blocker-based dual therapy.

**Methods:**

This 8-week, randomized, open-label, parallel-group, noninferiority study compared once-daily evening dosing of LTFC with the unfixed combination of latanoprost, one drop in the evening, and timolol, one drop in the morning (LTuFC). The primary efficacy endpoint was the mean change from baseline to week 8 in diurnal intraocular pressure (IOP; mean of 8 AM, 10 AM, 2 PM, 4 PM IOPs). LTFC was considered noninferior to LTuFC if the upper limit of the 95% confidence interval (CI) of the difference was < 1.5 mmHg (analysis of covariance).

**Results:**

Baseline characteristics were similar for LTFC (N = 125; POAG, 70%; mean IOP, 25.8 mmHg) and LTuFC (N = 125; POAG, 69%; mean IOP, 26.0 mmHg). Mean diurnal IOP changes from baseline to week 8 were -8.6 mmHg with LTFC and -8.9 mmHg with LTuFC (between-treatment difference: 0.3 mmHg; 95%-CI, -0.3 to 1.0). Both treatments were well tolerated.

**Conclusions:**

A single evening dose of LTFC was at least as effective as the unfixed combination of latanoprost in the PM and timolol in the AM in reducing IOP in Chinese subjects with POAG or OH whose IOP was insufficiently reduced with β-blocker monotherapy or β-blocker-based dual therapy. LTFC is an effective and well tolerated once-daily treatment for POAG and OH.

**Trial registration:**

Clinicaltrials.gov registration: NCT00219596

## Background

In China, 158 million people are over the age of 60, with the number projected to increase to approximately 250 million by the year 2020 [1]. As the median age rapidly rises, age-related diseases of the eye are emerging as a major public health issue. Primary open-angle glaucoma (POAG) in Chinese individuals over the age of 40 years has an estimated prevalence of approximately 1.5% to 2% [2]. Reliable data concerning the prevalence in China of ocular hypertension (OH), characterized by intraocular pressure (IOP) > 21 mmHg without ocular nerve damage and visual field loss, are not available.

Monotherapy with β-adrenergic antagonists such as timolol 0.5%, which lowers IOP levels by reducing aqueous humor outflow [3-5], continues to be among the most widely used approaches to treating POAG and OH in China. Although timolol generally is well tolerated, a significant proportion of timolol-treated patients does not achieve targeted IOP levels [6], and approximately one-third require a change in or addition to initial timolol monotherapy after 1 year, a proportion that increases to one-half after 2 years [7].

Latanoprost 0.005%, a selective prostaglandin F_2α _receptor agonist that has a mechanism of action complementary to that of timolol (i.e., acts mainly by increasing outflow) [8], has been shown to be an effective treatment for POAG and OH as monotherapy [9-12]. Results of a cross-national meta-analysis suggested that there is an efficacy advantage for latanoprost compared to timolol in Chinese patients with POAG that is independent of other clinical and demographic variables [13].

Patients who do not achieve target IOP levels with a single ocular hypotensive agent often are prescribed concomitant therapy with a medication that has a different mechanism of action, an approach supported by the American Academy of Ophthalmology [14] and the European Glaucoma Society [15]. In patients with POAG or OH whose IOP is not sufficiently controlled on timolol monotherapy, concomitant treatment with latanoprost has demonstrated additive IOP-reducing efficacy [10,16-18]. Moreover, in a double-masked comparison, evening dosing of fixed-combination latanoprost/timolol (Xalacom^®^; LTFC) was found to be at least as effective as latanoprost instilled once daily in the evening and timolol administered in both the morning and evening [19].

The purpose of the current study in Chinese patients was to evaluate the efficacy and tolerability of a single evening dose of LTFC versus the unfixed combination of latanoprost administered once daily in the evening and timolol dosed once daily in the morning (LTuFC).

## Methods

### Study design

This was an 8-week, randomized, open-label, parallel-group study conducted at 8 sites in China between June 30, 2005, and September 13, 2006 (NCT00219596). The study was conducted in accordance with the tenets of the Declaration of Helsinki and with all Chinese regulatory requirements and was approved by the Ethics Committee of Peking Union Medical College Hospital, Beijing, China. Written informed consent was obtained from study participants prior to study entry.

### Subjects

Males or females were eligible if they were 18 years of age or older, were diagnosed with POAG or OH and had been treated for at least the 4 weeks immediately prior to screening with β-blocker monotherapy or with a β-blocker-based dual therapy. At the screening visit, the IOP was required to be ≥17 and ≤35 mmHg in at least 1 eye at both the 8 AM and 10 AM measurement time points. At the baseline visit after washout (described below), IOP measured at 10 AM was required to be ≥21 and ≤35 mmHg and ≥25% higher than the IOP level at screening in at least 1 eye (same eye at both time points) for β-blocker monotherapy users, or ≥21 and ≤35 mmHg and ≥30% higher than the IOP at screening in at least 1 eye (same eye at both time points) for those treated with β-blocker-based dual therapy.

Potential subjects were excluded if they met any of the following criteria: (1) closed/barely open anterior chamber angle or a history of acute angle-closure glaucoma; (2) ocular surgery in one or both eyes within 3 months prior to the screening visit; (3) any condition in one or both eyes that could prevent reliable applanation tonometry; (4) ocular inflammation/infection occurring within 1 month prior to the screening visit; (5) use, or planned use, of any topical or systemic medication known to affect IOP; (6) known hypersensitivity to benzalkonium chloride or to any component of the study drug solutions; (7) any ocular or medical condition in which treatment with β-blocking agents is contraindicated; and (8) any acute or uncontrolled medical or psychiatric illness. Pregnant or lactating women or women of childbearing potential not using an acceptable method of contraception also were excluded.

### Treatments and Assessments

After initial screening, subjects completed a washout period that varied in length depending on the current drug as follows: 4 weeks for β-adrenergic antagonists and prostaglandin analogs (including latanoprost, bimatoprost, travoprost, and unoprostone); 2 weeks for adrenergic agonists; and 5 days for cholinergic agonists and carbonic anhydrase inhibitors.

After the washout period, all eligible subjects were assigned a randomization number by the site coordinator. Subjects were randomized in a 1:1 ratio according to a computer-generated pseudorandomization number generator using random permuted blocks with a fixed block size to the LTFC or LTuFC group. Each site was provided with sealed envelopes marked with sequential randomization numbers containing the name of the treatment to which the subject was randomized. Envelopes were returned to the sponsor at study completion. Randomized subjects received either one drop of LTFC at approximately 8 PM or the LTuFC consisting of one drop of latanoprost 0.005% at approximately 8 PM and one drop of timolol 0.5% at approximately 8 AM. At the baseline visit, subjects received study medication sufficient for 4 weeks; at the week 4 visit, medication sufficient for the remaining 4 weeks of the study was dispensed. Postbaseline study visits occurred at weeks 1, 2, 4, 6, and 8. Study medication was discontinued at week 8 whereupon subjects continued therapy with available medication. Subjects had a final follow-up visit at week 10.

IOP was measured at 8 AM and 10 AM at screening; at 8 AM, 10 AM, 2 PM, and 4 PM at baseline and week 8 (or early discontinuation); and at 8 AM only at weeks 1, 2, 4, and 6. At each measurement time point, two assessments were performed in each eye, alternating between eyes, and the IOP at a given time point was defined as the average of the two measures. For each subject, IOP assessments were made by the same examiner using the same calibrated Goldmann applanation tonometer at each visit. Additional evaluations performed at each visit included assessment of systemic and ocular adverse events; recording of vital signs; visual acuity measurement; and lid, slit lamp, and ophthalmoscopy examinations of eye structures. Visual field examinations were performed at screening and week 8.

### Endpoints and analyses

The primary efficacy endpoint was change from baseline in mean diurnal IOP at week 8. If only one eye was eligible, the diurnal IOP for each measurement day was the mean of IOP measurements at 8 AM, 10 AM, 2 PM, and 4 PM of the study eye. If both eyes were eligible, the diurnal IOP for each measurement day was calculated as the mean of diurnal IOP levels across study eyes.

The study was designed to evaluate whether LTFC was noninferior to LTuFC, i.e., was either more or similarly effective. Between-group differences were evaluated using an analysis of covariance (ANCOVA) model with baseline diurnal IOP as a covariate and treatment and center as factors. The treatment difference (ΔLTFC - ΔLTuFC) and the corresponding 95% confidence interval (CI) for the difference were calculated. LTFC was considered noninferior to LTuFC if the upper limit of the 2-sided 95% CI of the difference was < 1.5 mmHg. A sample of 100 subjects per treatment group was estimated to provide at least 85% power using a 1-sided test with a 2.5% significance level.

For secondary efficacy endpoints, the statistical significance of between-group differences in mean 8 AM IOP at weeks 1, 2, 4, 6, and 8 and mean change in IOP from baseline to week 8 at each measurement time point were analyzed using the ANCOVA method described with regard to the primary endpoint. Between-group differences in proportions of subjects reaching prespecified diurnal IOP levels of ≤21, ≤18, ≤16, and ≤14 mmHg at week 8 were evaluated using the Cochran-Mantel-Haenszel test.

Intent-to-treat (ITT) efficacy analyses included all subjects who received study medication, had an IOP measurement at baseline, and had at least one valid postbaseline IOP measurement. Missing efficacy data were imputed using the last observation carried forward (LOCF) method; baseline values were not carried forward.

## Results

**Figure 1:**
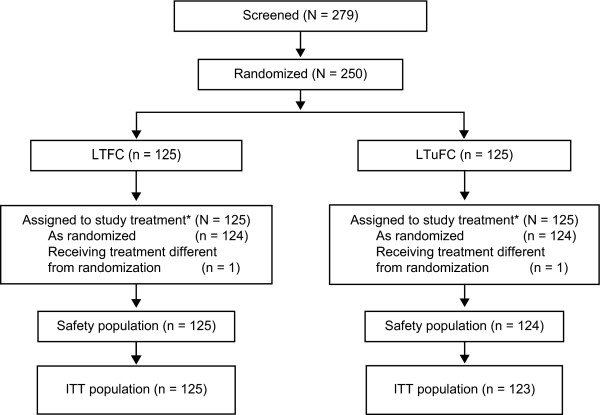
**Analysis population classification**. LTFC = latanoprost/timolol fixed combination; ITT = intent-to-treat population; LTuFC = latanoprost/timolol unfixed combination.

In all, 279 subjects were screened, and 250 completed the 4-week washout period, continued to meet eligibility criteria, and were randomized to study treatment (Figure 1). In the LTFC group, both the safety and ITT populations included the 125 subjects who received treatment. In the LTuFC group, one subject did not receive study medication and one did not have required IOP measurements; the safety and ITT populations for this group included 124 and 123 subjects, respectively. In both treatment groups, 120 of 125 screened subjects (96%) completed the study.

Subjects' demographic and baseline clinical characteristics are summarized in Table 1. With the exception of a somewhat greater proportion of females in the LTFC group (53% vs. 40%, respectively), treatment groups were similar with regard to age, diagnosis, mean baseline IOP, and prior ocular hypotensive medication. Four subjects (3.2%) in each treatment group had a history of diabetes mellitus; 16 (12.8%) subjects in the LTFC and 12 (9.6%) in the LTuFC group had a history of hypertension.

**Table 1:** Subject characteristics*

	LTFC N = 125	LTuFC N = 124
Female, n (%)	66 (52.8)	50 (40.3)
Age, years, mean (range)	50.0 (18-75)	47.9 (17-74)
Primary open-angle glaucoma, n (%)	87 (69.6)	85 (68.5)
Duration since diagnosis, years, mean (range)	4.2 (0.1-30)	3.1 (0.1-33)
Ocular hypertension, n (%)	38 (30.4)	39 (31.5)
Duration since diagnosis, years, mean (range)	2.7 (0.2-25)	2.3 (0.2-10)
Baseline IOP, mmHg, mean ± SD^†^	25.8 ± 3.2	26.0 ± 3.5
Prior medications in ≥10% of subjects, n (%)		
Brinzolamide	101 (80.8)	93 (75.0)
Carteolol hydrochloride	49 (39.2)	42 (33.9)
Timolol maleate	28 (22.4)	33 (26.6)
Timolol	22 (17.6)	24 (19.4)
Carteolol	15 (12.0)	13 (10.5)

*Safety population.

^†^Includes subjects with data at both baseline and week 8; N = 123 for the LTuFC group.

LTFC = latanoprost/timolol fixed combination; IOP = intraocular pressure; SD = standard deviation; LTuFC = latanoprost/timolol unfixed combination.

### Efficacy

At baseline, mean diurnal IOP levels were approximately 26 mmHg in both treatment groups (Table 2). Mean IOP reductions of > 8 mmHg from baseline to week 8 were observed in both groups (P < 0.001 for each group). The least square mean reduction in diurnal IOP from baseline to week 8 was 8.6 mmHg in LTFC-treated subjects and 8.9 mmHg among those treated with LTuFC. The between-group difference was 0.3 mmHg (95% CI: -0.3, 1.0); the upper bound of the 95% CI was < 1.5 mmHg, indicating noninferiority of LTFC.

**Table 2:** Change in diurnal IOP pressure (mmHg) at week 8 (primary endpoint)*

	LTFC N = 125	LTuFC N = 123
Baseline, mean ± SD	25.8 ± 3.2	26.0 ± 3.5
Week 8, mean ± SD	17.5 ± 2.9	17.2 ± 3.0
Change from baseline to week 8, mean ± SD	-8.3 ± 3.2	-8.8 ± 3.8
(95% CI)	(-8.9 to -7.7)	(-9.5 to -8.1)
LS mean change	-8.6	-8.9
Difference in LS means (95% CI)	0.3 (-0.3 to 1.0)	

*Intent-to-treat population.

CI = confidence interval; LTFC = latanoprost/timolol fixed combination; IOP = intraocular pressure; LS = least square; SD = standard deviation; LTuFC = latanoprost/timolol unfixed combination.

**Figure 2:**
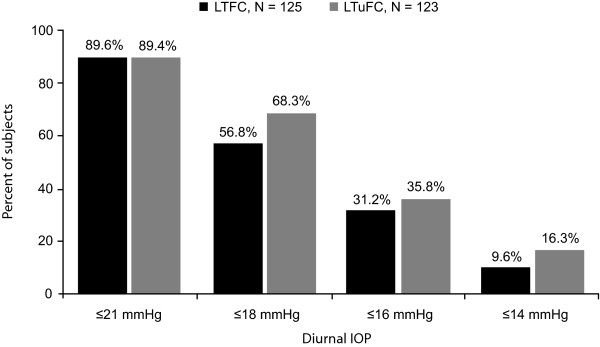
Proportion of subjects achieving prespecified IOP levels at week 8*. *Intent-to-treat population. No significant between-group differences based on the Cochran-Mantel-Haenszel test. LTFC = latanoprost/timolol fixed combination; IOP = intraocular pressure; LTuFC = latanoprost/timolol unfixed combination.

No statistically significant between-group differences were noted in proportions of subjects reaching prespecified percentage mean diurnal IOP levels at week 8 (Figure 2).

**Figure 3:**
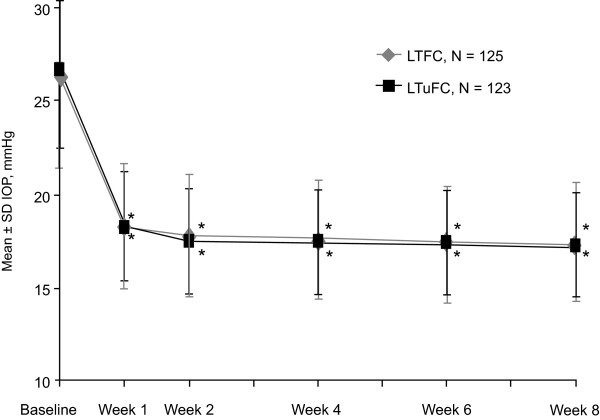
Mean 8 AM IOP across 8 weeks of treatment*. *Intent-to-treat population. P < 0.001 for baseline to postbaseline treatment difference based on analysis of covariance with treatment and center as factors and baseline 8 AM IOP as a covariate. LTFC = latanoprost/timolol fixed combination; IOP = intraocular pressure; LTuFC = latanoprost/timolol unfixed combination.

**Figure 4:**
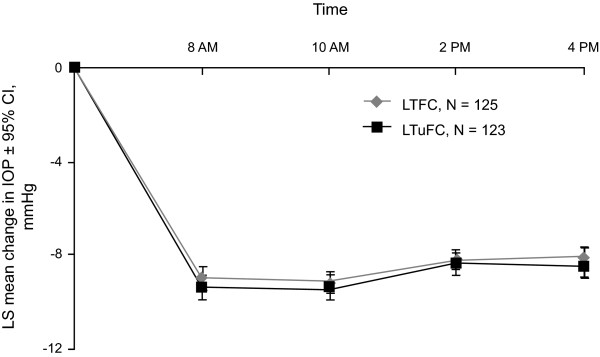
Mean change in IOP from baseline to week 8 at each measurement time point*. *Intent-to-treat population. P > 0.17 for each between-treatment difference based on analysis of covariance with treatment and center as factors and baseline diurnal IOP as a covariate. LTFC = latanoprost/timolol fixed combination; IOP = intraocular pressure; LS = least square; LTuFC = latanoprost/timolol unfixed combination.

The IOP-reducing effect of both the fixed and unfixed combinations was evident at week 1 and was sustained through week 8. Mean 8 AM IOP levels were reduced significantly (P < 0.001) from baseline at weeks 1, 2, 4, 6, and 8 in both treatment groups, and no between-groups difference in reduction was statistically significant (Figure 3). At week 8, least square mean IOP reductions from baseline were similar across treatments at all measurement time points (Figure 4).

### Adverse events

The incidence of all-causality treatment-emergent adverse events was low in both treatment groups, with 11.2% of those receiving LTFC and 6.5% of subjects treated with LTuFC reporting at least one such event (Table 3). No event in either group was considered by an investigator to be severe or serious. Conjunctival hyperemia was the only adverse event to occur in > 3% of subjects in either group and was reported in 7.2% of those treated with LTFC and 4.8% of subjects in the LTuFC group. Two subjects in each group discontinued due to an adverse event (LTFC: blurred vision of moderate severity, right bundle branch block; LTuFC: mild chest pain, viral conjunctivitis); one discontinuation in each group was considered to be treatment related (LTFC: blurred vision; LTuFC: mild chest pain). No clinically significant, treatment-emergent abnormalities in vital signs were noted in either treatment group during the study, and there were no clinically important between-group differences in ocular safety assessments or visual field examination findings.

**Table 3:** All-causality treatment-emergent adverse events, n (%)*

	LTFC N = 125	LTuFC N = 124
Subjects with ≥1 adverse event	14 (11.2)	8 (6.5)
Subjects with ≥1 severe adverse Event	0	0
Subjects with ≥1 serious adverse Event	0	0

Conjunctival hyperemia	9 (7.2)	6 (4.8)
Visual field defect (mild)	3 (2.4)	0
Eye irritation	1 (0.8)	0
Eyelid cyst	1 (0.8)	0
Eyelid disorder	1 (0.8)	0
Vision blurred	1 (0.8)	0
Right bundle branch block	1 (0.8)	0
Chest pain	0	1 (0.8)
Viral conjunctivitis	0	1 (0.8)

*Safety population.

LTFC = latanoprost/timolol fixed combination; LTuFC = latanoprost/timolol unfixed combination.

## Discussion

In patients needing two drugs to achieve therapeutic goals, fixed-combination agents such as LTFC may have advantages over the multiple ocular hypotensives instilled at different times of day. In particular, compliance may be improved with the convenience of once-daily dosing, an important consideration since medication compliance among patients with POAG and OH has been shown to be reduced with more complex medication regimens [20-24], and poor adherence has been associated with vision decreases in glaucoma patients [25,26]. In addition, once-daily dosing may result in cost savings as well as less exposure to preservatives such as purite, sofZia (Alcon, Fort Worth, Texas), or benzalkonium chloride [27,28].

This 8-week, randomized, open-label study in Chinese patients with POAG or OH whose IOP was insufficiently controlled on a β-blocker administered either as monotherapy or as part of dual therapy demonstrated that both once-daily LTFC administered in the evening and the unfixed combination of latanoprost dosed in the evening and timolol administered in the morning significantly lowered IOP levels from baseline. In both groups, mean IOP levels were reduced from 26 mmHg at baseline to approximately 17 mmHg at week 8. For the primary endpoint, the between-group difference in least square mean diurnal IOP reductions from baseline to week 8 was 0.3 mmHg and the upper limit of the 95% CI was < 1.5 mmHg supporting the noninferiority of LTFC. These findings and the conclusion of noninferiority are similar to results of a randomized, double-masked trial [19] in which mean diurnal IOP reductions from baseline to week 12 were 8.7 and 9.0 mmHg with LTFC and LTuFC (timolol dosed twice daily), respectively, and the between-treatment difference was 0.3 mmHg (95% CI: -0.1, 0.7 mmHg; P = 0.15). At last measurement (8 weeks herein and 12 weeks in Diestelhorst and Larsson [19]), approximately 90% of subjects in both treatment groups achieved mean diurnal IOP levels ≤21 mmHg, and > 30% had mean diurnal IOPs ≤16 mmHg.

A recent systematic review and meta-analysis of 29 randomized clinical trials of the IOP-lowering effect of prostaglandins combined with topical β-blockers [29] found equivalent IOP reductions with morning or evening instillation of fixed combinations of timolol and a prostaglandin analog. However, only 24-hour studies are appropriate when addressing morning versus evening administration, and several studies have shown that the relative efficacy of LTFC and latanoprost reflects instillation time. For example, a study by Alm et al [9] as well as several by Konstas et al [30-32] demonstrated that evening dosing of latanoprost and LTFC provided lower daytime IOP levels than morning dosing. A study by Konstas and associates [32] showed that LTFC compared to latanoprost monotherapy, both dosed in the evening, provided a wider margin (2.5 mmHg more than latanoprost) over 24 hours than the morning dosing used in a regulatory trial [33]. In another crossover study [34], 24-hour IOP fluctuation was significantly lower with LTFC dosed in the evening compared with timolol alone (3.2 mmHg vs 4.4 mmHg, respectively; P = 0.003). Finally, a direct 24-hour IOP comparison between morning and evening administration of LTFC in POAG patients found that evening dosing provided more effective IOP control [35].

In addition to the issue of administration time, no study of LTFC has included evenly distributed trough and peak measurements [29]. Moreover, although timolol administered once daily has been shown to achieve maximum IOP reduction [36], the impact of circadian rhythm remains to be clarified. Peak IOP values in the morning and nadir values in the evening have been reported for both healthy elderly volunteers [37] and for untreated individuals with glaucoma [38]. In the current study, IOP assessments were limited to the period between 8 AM and 4 PM, which coincided with the first 8 hours after the morning administration of timolol in the unfixed combination.

The meta-analysis [29] found that greater IOP lowering occurred with concomitant timolol twice daily and latanoprost once daily than with LTFC, a difference that may reflect the omission of a timolol dose with the fixed combination. Herein, timolol was administered once daily in the morning in the LTuFC arm rather than twice daily as is more typical with unfixed regimens. Instillation of one dose of timolol in the morning in both treatment arms may explain, in part, the relatively small between-group difference in mean IOP reduction.

Consistent with the results of previous studies [19,27,39-41], both the fixed and unfixed combinations of latanoprost/timolol were well tolerated. Rates of treatment-emergent adverse events were somewhat lower in the present study than reported by Diestelhorst and Larsson [19] possibly reflecting the difference in follow-up (8 weeks vs 12 weeks, respectively). In the present study, only one adverse event (conjunctival hyperemia) occurred in > 3% of subjects in each treatment group, and only two subjects in each group discontinued due to treatment-related adverse events. There were no clinically important between-group differences in treatment-emergent changes in ocular safety assessments. It is important to note, however, that the duration of the present study was too short to identify possible long-term adverse treatment effects.

This is the first study to compare the efficacy and tolerability of LTFC with that of LTuFC in a Chinese population, a population that represents an increasingly large proportion of individuals with POAG or OH worldwide. The study is limited by its open-label design. However, the randomization of subjects and the assessment of outcomes at multiple visits and the fact that our results closely parallel those of a prior double-masked study [19] suggest that the impact of the open-label design may have been minimal. The research also was limited by its short time frame since follow-up periods of several years would be needed to assess the progression of glaucomatous damage. Although Watson et al [12] found that POAG patients treated with latanoprost monotherapy experienced a significantly greater mean IOP reduction than similarly treated OH patients (9.4 mmHg vs 7.1 mmHg, respectively), such an analysis was not prespecified in the present study; future research might profitably compare differences between these diagnosis groups in Chinese patients.

## Conclusion

Single nighttime dosing with LTFC is well tolerated and at least as effective as concomitant administration of latanoprost and timolol each administered once daily. The benefits of combination treatment argue for its consideration by clinicians when two drugs are needed to meet therapeutic goals.

## Competing interests

Dr. Zheng Zhong is an employee of Pfizer Investment Co., Ltd., China. The other authors have no proprietary or commercial interest in any of the materials discussed in this article.

## Authors' contributions

JLZ was the principal investigator of the study. He made substantial contributions to the conception and design, acquisition of data, and analysis and interpretation of data for this study. He participated in drafting the article and in the critical revision of the manuscript for important intellectual content.

JG, XXL, YML, YHS, NXS, XHS, and KY were the principal investigators in each of the medical centers involved in the study. They participated in the design, acquisition and interpretation of data, and critical revision of the manuscript for important intellectual content. ZZ participated in the analysis and interpretation of data and in the critical revision of the manuscript for important intellectual content. All authors read and approved the final manuscript.

Members of the Xalacom Study Group in China participated in the study.

The results of this study were presented in part at the IIV Congress of the Asian-Oceanic Glaucoma Society and 2008 National Congress of the Chinese Glaucoma Society, Dec.5-7, 2008, Guangzhou, China.

## Pre-publication history

The pre-publication history for this paper can be accessed here:

http://www.biomedcentral.com/1471-2415/11/23/prepub
